# Evolutionary History of the Odd-Nosed Monkeys and the Phylogenetic Position of the Newly Described Myanmar Snub-Nosed Monkey *Rhinopithecus strykeri*

**DOI:** 10.1371/journal.pone.0037418

**Published:** 2012-05-16

**Authors:** Rasmus Liedigk, Mouyu Yang, Nina G. Jablonski, Frank Momberg, Thomas Geissmann, Ngwe Lwin, Tony Htin Hla, Zhijin Liu, Bruce Wong, Li Ming, Long Yongcheng, Ya-Ping Zhang, Tilo Nadler, Dietmar Zinner, Christian Roos

**Affiliations:** 1 Primate Genetics Laboratory, German Primate Center, Göttingen, Germany; 2 Fanjingshan National Nature Reserve, Jiangkou, Guizhou Province, China; 3 Department of Anthropology, The Pennsylvania State University, University Park, Pennsylvania, United States of America; 4 Fauna and Flora International (FFI), Myanmar Programme, Yangon, Myanmar; 5 Anthropological Institute, University Zürich-Irchel, Zürich, Switzerland; 6 Biodiversity and Nature Conservation Association (BANCA), Yangon, Myanmar; 7 Key Laboratory of Animal Ecology and Conservation Biology, Institute of Zoology, Chinese Academy of Sciences, Beijing, China; 8 The Nature Conservancy, Kunming, China; 9 Kunming Institute of Zoology, Chinese Academy of Sciences, Kunming, China; 10 Frankfurt Zoological Society, Endangered Primate Rescue Center, Cuc Phuong National Park, Ninh Binh Province, Vietnam; 11 Cognitive Ethology Laboratory, German Primate Center, Göttingen, Germany; 12 Gene Bank of Primates, German Primate Center, Göttingen, Germany; Texas A&M University, United States of America

## Abstract

Odd-nosed monkeys represent one of the two major groups of Asian colobines. Our knowledge about this primate group is still limited as it is highlighted by the recent discovery of a new species in Northern Myanmar. Although a common origin of the group is now widely accepted, the phylogenetic relationships among its genera and species, and the biogeographic processes leading to their current distribution are largely unknown. To address these issues, we have analyzed complete mitochondrial genomes and 12 nuclear loci, including one X chromosomal, six Y chromosomal and five autosomal loci, from all ten odd-nosed monkey species. The gene tree topologies and divergence age estimates derived from different markers were highly similar, but differed in placing various species or haplogroups within the genera *Rhinopithecus* and *Pygathrix*. Based on our data, *Rhinopithecus* represent the most basal lineage, and *Nasalis* and *Simias* form closely related sister taxa, suggesting a Northern origin of odd-nosed monkeys and a later invasion into Indochina and Sundaland. According to our divergence age estimates, the lineages leading to the genera *Rhinopithecus*, *Pygathrix* and *Nasalis*+*Simias* originated in the late Miocene, while differentiation events within these genera and also the split between *Nasalis* and *Simias* occurred in the Pleistocene. Observed gene tree discordances between mitochondrial and nuclear datasets, and paraphylies in the mitochondrial dataset for some species of the genera *Rhinopithecus* and *Pygathrix* suggest secondary gene flow after the taxa initially diverged. Most likely such events were triggered by dramatic changes in geology and climate within the region. Overall, our study provides the most comprehensive view on odd-nosed monkey evolution and emphasizes that data from differentially inherited markers are crucial to better understand evolutionary relationships and to trace secondary gene flow.

## Introduction

**Figure 1:**
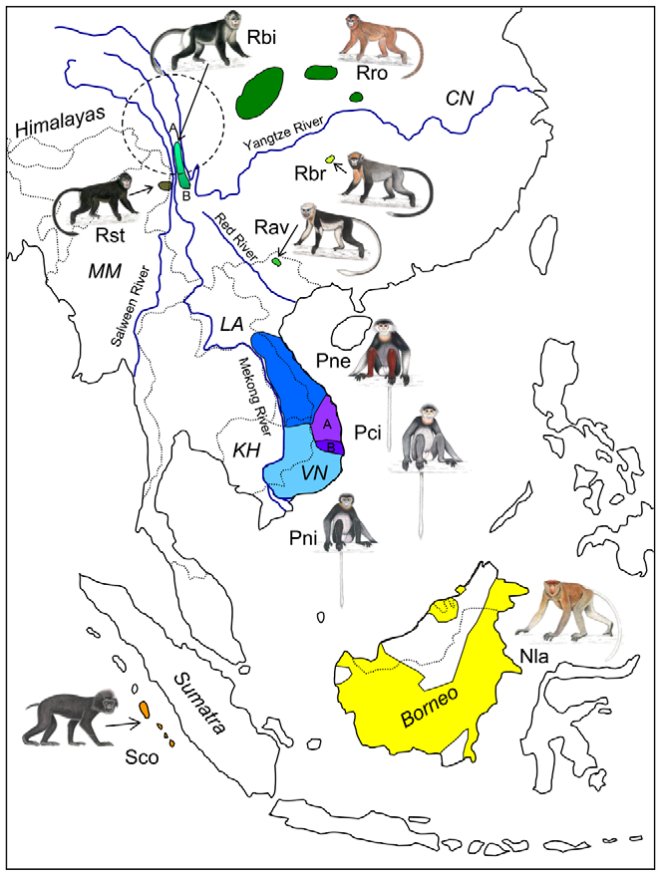
Idealized distribution map of the ten odd-nosed monkey species in South-east Asia. Hatched and blue lines indicate country borders and major rivers, and the Hengduan Mountain range as presumed center of origin is depicted as dashed circle. Abbreviations used in the figure: Rro = *R. roxellana*, Rbi = *R. bieti*, Rst = *R. strykeri*, Rbr = *R. brelichi*, Rav = *R. avunculus*, Pne = *P. nemaeus*, Pci = *P. cinerea*, Pni = *P. nigripes*, Sco = *S. concolor*, and Nla = *N. larvatus*, CN = China, KH = Cambodia, LA = Laos, MM = Myanmar, VN = Vietnam. A and B refer to the distribution of main haplogroups of *R. bieti* and *P. cinerea*, respectively. Illustrations by Stephen Nash, Conservation International.

The course of mammalian evolution in the Tertiary and Quaternary has been affected profoundly by changes in continental configuration, mountain building, and associated changes in patterns of oceanic and atmospheric circulation. Nowhere is this better illustrated than in eastern Eurasia during the late Tertiary (23.0–2.588 million years), where the raising of the Himalayas and adjacent Tibetan Plateau altered the biogeographic landscape by creating new physical and climatic barriers to gene flow. Environmental changes in the region of the Hengduan Mountains (including today's Three Parallel Rivers National Park and World Heritage Site, Figure 1) in South-western China had pronounced effects on biotas, creating opportunities for the preservation of paleoendemics and for the creation of neoendemics in sheltered, trenchant intermontane valleys [1], [2]. Tectonic activities, changes in global temperature, and the inauguration of Pleistocene glacial and interglacial fluctuations led to further cycles of dissection and coalescence of landscape and biotas. Heightened environmental seasonality and changes in sea level affecting the inundation and exposure of low-lying continental shelf environments, such as large parts of South-East Asia (e.g., Sundaland), created dynamic landscapes, which witnessed both the extinction and origination of numerous mammalian species. The primate order was no exception with radiations in gibbons, macaques, langurs and odd-nosed monkeys as well as extinctions, e.g. in the Pongidae (*Gigantopithecus* and *Pongo* on the Asian mainland) [3], [4]. In this geographical and historical context odd-nosed monkeys are of particular interest, because they occupy a large latitudinal and altitudinal range including their presumed centre of origin (Hengduan Mountains, Figure 1) [5].

Odd-nosed monkeys are enigmatic and rare, and they show remarkable anatomical and behavioral adaptations to a range of habitats unusual for primates, such as mangrove swamps, and temperate and high altitude forests [5], [6]. The group consists of four genera, snub-nosed monkeys (*Rhinopithecus*), douc langurs (*Pygathrix*), proboscis monkeys (*Nasalis*) and pig-tailed monkeys (*Simias*). Three of the five snub-nosed monkey species, *R. roxellana*, *R. brelichi* and *R. bieti* are endemic to China, while a fourth species, *R. avunculus* is restricted to the North of Vietnam. A fifth species, *R. strykeri* was recently described from Myanmar (Burma) [7], and has now also been confirmed in Nujiang prefecture, China, in contiguous forests with Myanmar. Douc langurs, represented by the three species *P. nemaeus*, *P. cinerea* and *P. nigripes*, are distributed through parts of Vietnam, Laos and Cambodia, east of the Mekong River. Both *Nasalis* and *Simias* are monotypic. *N. larvatus* is a pure Bornean species, whereas *S. concolor* is endemic to the Mentawai Islands, west off Sumatra. All species are endangered or critically endangered [7], [8]. *R. avunculus* and *R. strykeri* are the most threatened species with populations sizes of ∼250 and 260–330 individuals, respectively, followed by *P. cinerea* (550–700 individuals) and *R. brelichi* (∼750 individuals). *R. bieti* has less than 2,000 individuals, and *R. roxellana* and *S. concolor* have ∼15,000 and 6,700–17,300 individuals. For *P. nemaeus*, *P. nigripes* and *N. larvatus* no reliable estimates are available [7], [8].

Odd-nosed monkeys together with langurs, including the genera *Presbytis*, *Semnopithecus* and *Trachypithecus*, form the Asian colobines [5], [9]–[14]. Recent genetic investigations convincingly confirmed a common origin of the odd-nosed monkeys [11]–[16] and resolved the phylogenetic relationships among genera [14].

Within genera, a clearly resolved branching pattern was obtained for *Pygathrix* [17]–[19], but relationships among the *Rhinopithecus* species are still disputed. Jablonski and Peng [9] found that *R. roxellana* groups together with the *R. bieti*+*R. brelichi* clade and that *R. avunculus* forms a sister species to all other snub-nosed monkeys. Molecular studies based only short fragments of the mitochondrial genome suggested an unresolved polytomy [18]–[21], while in a recent study by Li et al. [15] using complete mitochondrial genome data, *R. avunculus* appears as sister lineage to a *R. bieti*+*R. roxellana* clade. Unfortunately, all previous molecular studies lack a complete taxon sampling. In particular, *R. strykeri* was not studied so far, but it can be assumed that this species is closely related to *R. bieti* [7].

Although our understanding of the phylogenetic relationships within the odd-nosed monkey group became clearer in recent years, several questions remain. All molecular studies so far used mainly only short mitochondrial fragments and/or did not include all species. To obtain a more complete picture about the evolutionary history of the odd-nosed monkeys, we have analyzed complete mitochondrial genomes (mtDNA) and 12 nuclear loci (nucDNA, five autosomal, six Y chromosomal, one X chromosomal) from all ten odd-nosed monkey species. Moreover, we included data from the two known major haplogroups of *R. bieti* [20], [22], [23] and *P. cinerea* (Roos unpublished). As a result of our study, we present here the most complete and updated molecular phylogeny of odd-nosed monkeys and discuss their phylogeographic implications.

## Results

### Mitochondrial phylogeny

**Figure 2:**
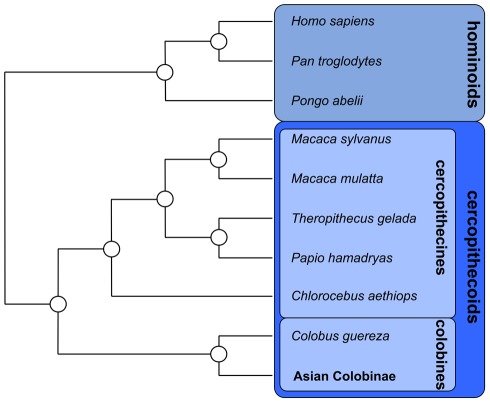
Phylogenetic relationships among studied non-Asian colobine species based on mitochondrial and nuclear datasets. All relationships are significantly supported by ML bootstrap values of 100% and posterior probabilities of 1.0 in all datasets (mitochondrial and nuclear datasets not combined).

**Figure 3:**
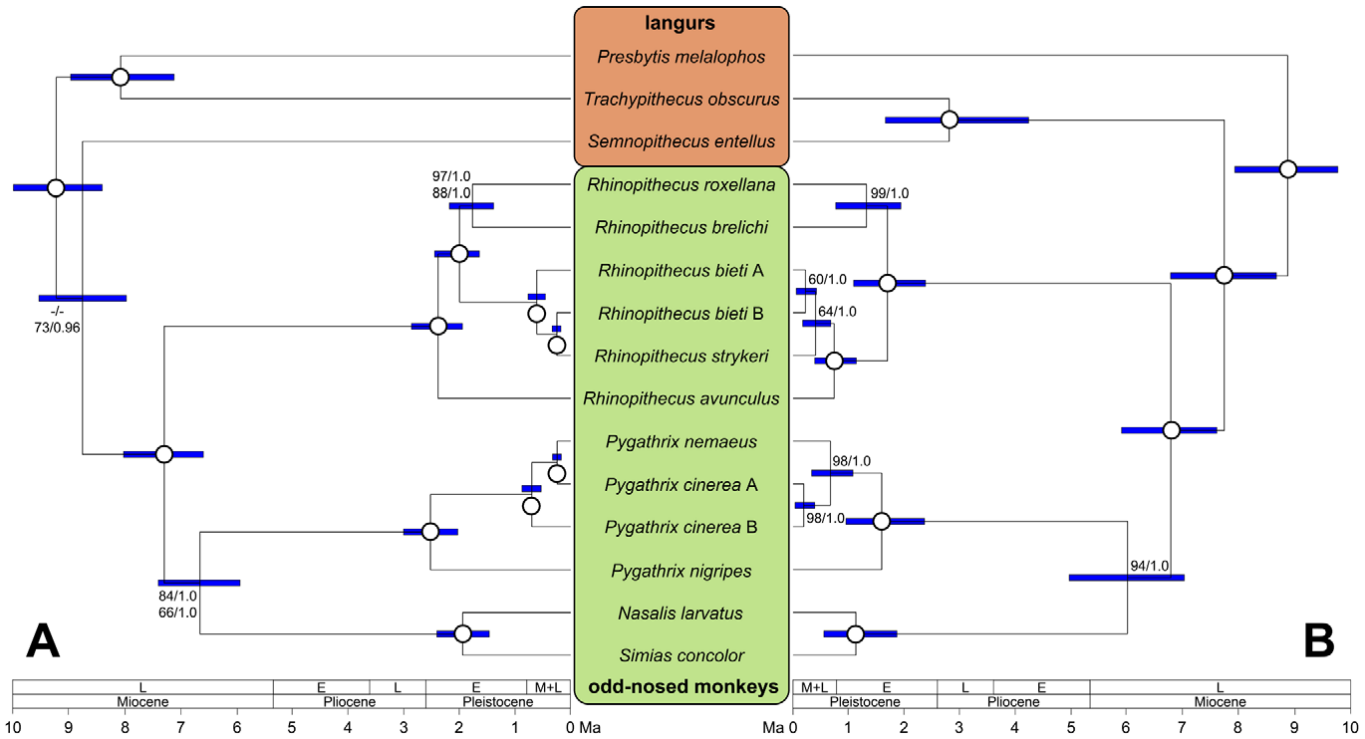
Ultrametric tree showing phylogenetic relationships among Asian colobines as obtained from mitochondrial (A) and nuclear sequence data (B). Open circles indicate ML bootstrap values of 100% and posterior probabilities of 1.0; values below are given at respective branches. Blue bars represent 95% highest posterior densities of divergence ages. In A, upper and lower numbers on branches indicate ML bootstrap values and posterior probabilities as derived from datasets mtDNA1 and mtDNA2, respectively. Abbreviations used in the bars: L = late, E = early, and M = middle.

The mtDNA alignment including a total of 24 primate sequences had a length of 16,920 bp (Table S1). After excluding indels and poorly aligned positions, 15,617 bp remained in the mtDNA1 dataset (for further details see Materials and Methods). Another alignment including only the 12 protein-coding genes on the heavy strand (mtDNA2) consisted of 10,851 bp. Phylogenetic relationships as obtained from maximum-likelihood (ML) and Bayesian reconstructions resulted in identical and mainly significantly supported branching patterns (Figure 2, Figure 3A). Asian colobines initially split into the three lineages/clades *Trachypithecus+Presbytis*, *Semnopithecus* and odd-nosed monkeys. While reconstructions based on the mtDNA1 dataset allowed no resolution of this trichotomy, the mtDNA2 dataset suggested a sister grouping of *Semnopithecus* with odd-nosed monkeys. Among odd-nosed monkeys, *Rhinopithecus* separated 7.28 million years ago (Ma) (for 95% highest posterior probabilities see Table 1), before also *Pygathrix* diverged 6.63 Ma from the *Nasalis*+*Simias* clade. Within *Rhinopithecus*, *R. avunculus* formed a sister lineage to the remaining species, which further divided into a clade consisting of *R. roxellana* and *R. brelichi*, and a clade with *R. bieti* and *R. strykeri*. Interestingly, *R. bieti* appeared paraphyletic with haplogroup B being more closely related to *R. strykeri* than to the con-specific haplogroup A. Within *Pygathrix*, *P. nigripes* separated first and haplogroup B of *P. cinerea* formed a sister lineage to a clade consisting of the *P. cinerea* haplogroup A and *P. nemaeus*. Initial splits in *Rhinopithecus* and *Pygathrix*, and the divergence of *Nasalis* and *Simias* occurred 2.50–1.75 Ma. Haplogroup A of *R. bieti* separated from haplogroup B and *R. strykeri* 0.60 Ma, while the latter two diverged 0.24 Ma. Haplogroup B of *P. cinerea* split from haplogroup A and *P. nemaeus* 0.69 Ma, and the latter two separated 0.23 Ma. According to alternative tree topology tests, alternative phylogenetic relationships among odd-nosed monkey species as suggested by nucDNA (Figure 3B) were rejected (P<0.001).

**Table 1:** Divergence ages in Ma (95% highest posterior density).

Split	mtDNAdivergence ages	nucDNAdivergence ages
Cercopithecoidea−Hominoidea	27.28 (24.93–29.48)	27.15 (24.44–29.68)
*Pongo−Homo+Pan*	13.65 (12.56–14.70)	14.07 (12.96–15.15)
*Homo−Pan*	6.17 (5.58–6.78)	6.36 (5.75–6.91)
Cercopithecinae−Colobinae	19.13 (16.92–21.65)	18.86 (15.21–22.61)
*Chlorocebus*−Papionini	12.16 (10.38–13.79)	11.54 (9.19–14.02)
*Macaca−Papio+Theropithecus*	9.94 (8.53–11.29)	8.36 (6.84–10.04)
*Macaca sylvanus−M. mulatta*	5.49 (4.97–6.02)	5.26 (4.68–5.84)
*Papio−Theropithecus*	4.07 (3.56–4.60)	3.85 (3.31–4.43)
*Colobus*−Asian Colobinae	12.25 (10.81–13.79)	11.84 (9.95–14.21)
*Presbytis+Trachypithecus−Semnopithecus*+odd-nosed monkeys	9.21 (8.39–9.98)	-
*Presbytis−Trachypithecus*	8.06 (7.10–8.95)	-
*Semnopithecus* – odd-nosed monkeys	8.74 (7.95–9.52)	-
*Presbytis* – other Asian Colobinae	-	8.86 (7.91–9.76)
*Semnopithecus+Trachypithecus* – odd-nosed monkeys	-	7.72 (6.76–8.66)
*Semnopithecus−Trachypithecus*	-	2.79 (1.65–4.22)
*Rhinopithecus* – other odd-nosed monkeys	7.28 (6.57–8.00)	6.77 (5.88–7.59)
*Pygathrix−Nasalis+Simias*	6.63 (5.92–7.38)	5.99 (4.94–7.01)
*Nasalis−Simias*	1.92 (1.45–2.39)	1.12 (0.54–1.85)
*Rhinopithecus avunculus* – other *Rhinopithecus* spp.	2.37 (1.93–2.84)	-
*R. roxellana+R. brelichi−R. bieti+R. strykeri*	1.99 (1.63–2.43)	-
*R. roxellana−R. brelichi*	1.75 (1.37–2.16)	1.31 (0.76–1.93)
*R. bieti* A*−*R. bieti* B+*R. strykeri*	0.60 (0.45–0.75)	-
*R. bieti* B−*R. strykeri*	0.24 (0.17–0.32)	-
*R. roxellana+R. brelichi* – other *Rhinopithecus* spp.	-	1.69 (1.08–2.37)
*R. avunculus−R. bieti+R. strykeri*	-	0.73 (0.38–1.13)
*R. strykeri−R. bieti*	-	0.40 (0.17–0.67)
*R. bieti* A−*R. bieti* B	-	0.22 (0.05–0.41)
*Pygathrix nigripes−P. cinerea+P. nemaeus*	2.50 (2.02–2.99)	1.58 (0.94–2.35)
*P. cinerea* B−*P. cinerea* A+*P. nemaeus*	0.69 (0.52–0.86)	-
*P. cinerea* A−*P. nemaeus*	0.23 (0.16–0.32)	-
*P. cinerea−P. nemaeus*	-	0.66 (0.32–1.07)
*P. cinerea* A−*P. cinerea* B	-	0.18 (0.03–0.38)

* A and B refer to the two major haplogroups in *R. bieti* and *P. cinerea*, respectively.

### Nuclear phylogeny

For phylogenetic analysis, nuclear sequence data were combined, because single loci provided only limited resolution in an earlier study [14] and partition homogeneity tests revealed no significant difference in their evolutionary history (Y chromosomal loci combined: P = 0.945; autosomal loci combined: P = 0.066; all nuclear loci combined: P = 0.200). The concatenated nuclear alignment had an original length of 15,101 bp (Table S1), but was reduced to 13,102 bp after indels and poorly aligned positions were removed. Phylogenetic relationships among Asian colobines were mainly significantly supported (Figure 3B), but partially disagreed with the mitochondrial topology. Notably, phylogenetic relationships as suggested by mtDNA (Figure 3A) were rejected (P = 0.002) by alternative tree topology tests. Differences to the mitochondrial phylogeny include the basal position of *Presbytis* among Asian colobines, the sister grouping of *Semnopithecus* and *Trachypithecus*, and species/haplogroup relationships within *Rhinopithecus* and *Pygathrix*. In *Rhinopithecus*, an initial split occurred between the *R. roxellana+R. brelichi* clade and the remaining taxa, including *R. avunculus*. Among the latter, *R. avunculus* diverged first and *R. strykeri* is basal to a monophyletic *R. bieti* clade. Within *Pygathrix*, *P. nigripes* represented the first split and *P. nemaeus* formed a sister lineage to the monophyletic *P. cinerea* clade. Calculated divergence ages for odd-nosed monkey genera and species were similar to the mtDNA estimates (Table 1). Accordingly, *Rhinopithecus*, *Pygathrix* and the *Nasalis+Simias* clade separated 6.77–5.99 Ma, and the latter split 1.12 Ma. Initial differentiation of *Rhinopithecus* (*R. roxellana+R. brelichi* – other *Rhinopithecus* spp., *R. roxellana−R. brelichi*) and *Pygathrix* (*P. nigripes* – other *Pygathrix* spp.) occurred on a similar time scale (1.69–1.31 Ma). Further splitting events in both genera took place 0.73–0.40 Ma (*R. avunculus−R. bieti+R. strykeri, R. bieti−R. strykeri*) and 0.66 Ma (*P. cinerea−P. nemaeus*), respectively.

## Discussion

As in earlier molecular studies including extended sequence data [11]–[16], the monophyly of the odd-nosed monkeys is strongly supported. Further, our data clearly suggests a basal position of *Rhinopithecus* and a sister grouping of *Pygathrix* with the *Nasalis*+*Simias* clade, a pattern also supported by two SINE integrations [14], and earlier nuclear [14], [16] and mitochondrial genome data [14]. Moreover, our study provides information concerning the branching pattern within the two polytypic genera *Rhinopithecus* and *Pygathrix*. In all gene trees, *R. roxellana* and *R. brelichi* form a monophyletic clade and *P. nigripes* is basal within *Pygathrix*. For all other *Rhinopithecus* and *Pygathrix* taxa, incongruent tree topologies were obtained. In *Rhinopithecus*, *R. avunculus* appears to be basal in the mtDNA tree, but in the nucDNA tree, the species is connected with the *R. bieti*+*R. strykeri* clade. The mtDNA tree further suggests paraphyly of *R. bieti*, but monophyly in the nucDNA tree. Similarly, within *Pygathrix*, *P. cinerea* is paraphyletic in the mtDNA tree, but monophyletic in the nucDNA tree.

Various reasons for incongruent gene tree topologies are possible [24], [25]. Hybridization (i.e., secondary gene flow) is one reason that becomes more and more accepted and recent studies show that hybridization occurs more frequently than previously thought in a broad range of species. Also for primates, hybridization was confirmed for almost all major radiations [26], [27]. In many cases, phylogenetic incongruences are caused by sex-biased introgessive hybridization which can occur if a secondary contact between two populations arises, migration is either male- or female-biased, and hybridization is unidirectional [28]–[30].

For odd-nosed monkeys, secondary gene flow seems to be also the most likely explanation for the observed incongruent gene tree topologies, because geographically close-by taxa cluster together. However, low resolution of phylogenetic relationships among species due to limited informative sites and incomplete lineage sorting might have an effect as well, in particular for nucDNA.

Assuming secondary gene flow as main reason for the observed gene tree discordances among odd-nosed monkey species and by incorporating estimated divergence ages, *R. avunculus* diverged first in *Rhinopithecus*, but came into secondary contact with the progenitor of *R. bieti* and *R. strykeri*. The progenitor of the latter two originally split into three major lineages (*R. bieti* haplogroup A, *R. bieti* haplogroup B, *R. strykeri*), with the *R. bieti* haplogroup B being closely related to *R. strykeri*. Interestingly, the *R. bieti* haplogroup B occurs more in the southern part of the species' range [22] and thus geographically closest to *R. strykeri*. Both *R. bieti* haplogroups came into contact again and homogenized their nuclear gene pools [31]. Today, both mitochondrial haplogroups of *R. bieti* intergrade, although there is still a trend of a northern and southern clade [22]. In *Pygathrix*, originally four major lineages emerged (*P. nigripes*, *P. cinerea* haplogroup A, *P. cinerea* haplogroup B, *P. nemaeus*), but secondary exchange of nuclear genes occurred between both haplogroups of *P. cinerea* and of mitochondria between *P. cinerea* haplogroup A and *P. nemaeus*. Both mitochondrial haplogroups of *P. cinerea* are today still geographically separated (Roos unpublished).

**Figure 4:**
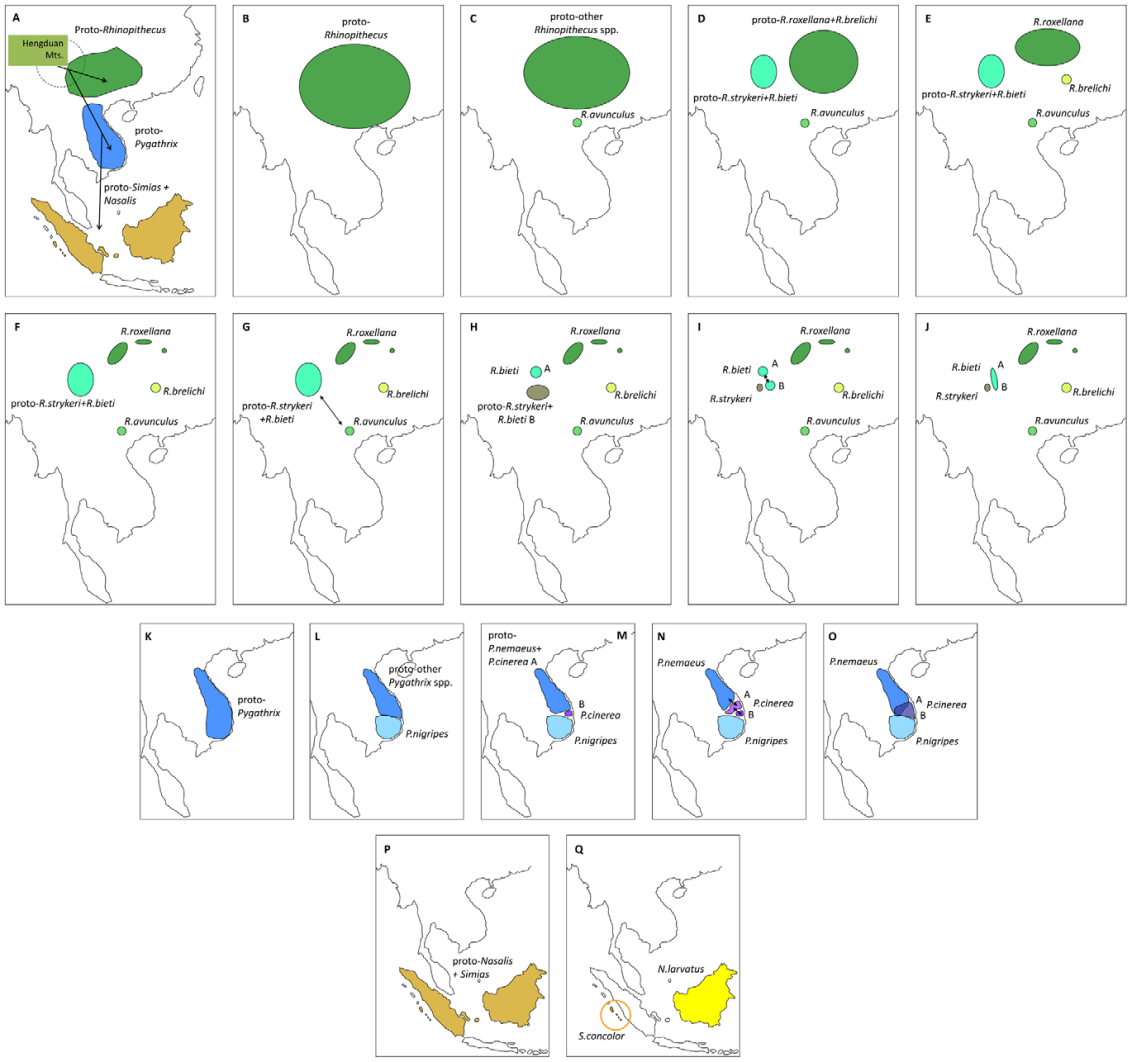
Proposed dispersal scenario for odd-nosed monkeys. (A) Odd-nosed monkeys most likely originated in the Hengduan Mountain range on the Asian mainland and migrated successively into Indochina and Sundaland during the late Miocene. In *Rhinopithecus* (B–J) and *Pygathrix* (K–O), differentiation into species started in the early Pleistocene, but secondary gene flow among various lineages occurred until the middle Pleistocene (indicated as arrows). (P, Q) In Sundaland, *Simias* and *Nasalis* split in the early Pleistocene. Most likely *Simias* or a common ancestor of *Simias* and *Nasalis* was also present on Sumatra, but became extinct there. A and B refer to the distribution of main haplogroups of *R. bieti* and *P. cinerea*, respectively. For further details see main text.

By combining the available information on phylogeny and past-geology and -climate in Asia, we develop the following dispersal scenario for odd-nosed monkeys (Figure 4). The origin of Asian colobine monkeys and also of the odd-nosed monkeys might have been the region of South-western China and the Hengduan Mountains in the border region of today's Myanmar, India and China [5], [14], [32]. The ancestor of the Asian colobines, including the odd-nosed monkeys, was probably a species of *Mesopithecus*, the colobine genus which was widespread in woodland and forested environments of eastern and southern Europe and western and southern Asia during the latest Miocene and earliest Pliocene [5], [33], [34]. The recent discovery of fossil colobine remains belonging to *Mesopithecus* in North-eastern Yunnan Province, near Zhaotong, supports this interpretation [35]. In the region, all the larger Southeast Asian rivers (Mekong, Salween, Yangtze) rise, which are all dispersal barriers for arboreal primates [36] at least since the early Miocene [37]. Habitat fragmentation became more pronounced in this region in the late Miocene as the result of regional tectonic uplift and subsidence related to the Himalayan orogeny [38]. After the separation of the langur progenitor from the odd-nosed monkey ancestor, members of the odd-nosed monkey group successively migrated from China to the South and expanded their range into Indochina and Sundaland in the latest Miocene. The migration into Sundaland by a progenitor of *Nasalis*+*Simias* was probably via land bridges connecting the mainland with Sundaland islands during periods of lowered sea levels [39]. Finally, *Nasalis* on Borneo and *Simias* on the Mentawai Islands diverged in the early Pleistocene. Due to the relatively prominent dating discrepancy, gene flow between both genera after the initial separation might have occurred, especially considering that migration was repeatedly possible via land bridge connections during the Pleistocene [39]. *Simias* or at least a common ancestor of *Simias* and *Nasalis* was most likely temporarily also present on Sumatra, but became extinct there of unknown reasons. Interestingly, the Mentawai archipelago shows a high rate of faunal and floral endemism and might have acted as refuge, as e.g. proposed for macaques [40]. In light of the prominent morphological differences between *Nasalis* and *Simias* the relatively late divergence is surprising and suggests that autapomorphic traits can emerge in a short time period.

Differentiation in *Rhinopithecus* started in the early Pleistocene, most likely triggered by reduction and fragmentation of suitable habitat. *Rhinopithecus* originally occupied a wide distribution, which became fragmented as the result of reduction of forest habitats resulting from climate/geological changes and changes in the course of rivers. Environmental changes occurring in the early Pleistocene appear to have produced recurrent episodes of habitat fragmentation and coalescence, allowing secondary contact and gene exchange. *R. avunculus* split off first, followed by the split between the northern species (*R. roxellana*, *R. brelichi*) and “Himalaya” species (*R. bieti*, *R. strykeri*). Shortly afterwards, *R. roxellana* and *R. brelichi* diverged and remained separated from each other. *R. avunculus* came into secondary contact with the progenitor of *R. bieti* and *R. strykeri* until the early middle Pleistocene. Subsequently, the ancestral *R. bieti+R. strykeri* population divided into two (*R. bieti* haplogroup A, *R. bieti* haplogroup B+*R. strykeri*) and later on into three populations (*R. bieti* haplogroup A, *R. bieti* haplogroup B, *R. strykeri*). *R. strykeri* remained afterwards separated from *R bieti*, while representatives of both *R. bieti* haplogroups came into secondary contact which lasts until today (as far as possible due to anthropogenic habitat fragmentation) [31]. The fossil record of *Rhinopithecus* broadly supports this interpretation. The genus enjoyed a wide distribution in central, eastern, and southern China in the Plio-Pleistocene [3], [41], [42], and even included a large-bodied extinct species, *R. lantianensis*, north of the Yellow River [43]. The extent of species differentiation in the Pleistocene is hard to determine from the fossil record, because most *Rhinopithecus* fossils are represented only by teeth and jaw fragments, and these exhibit only little diagnostic, species-specific morphological traits.

For *Pygathrix*, our genetic data suggest differentiation events on a similar time scale as in snub-nosed monkeys (see above), limestone langurs [16] and crested gibbons [44]. Thus, the same environmental changes as mentioned above might have influenced not only snub-nosed monkey differentiation, but also differentiation processes in douc langurs and other primates in the region. After the split of *P. nigripes*, the ancestor of *P. nemaeus* and *P. cinerea* successively diverged in the middle Pleistocene, leading first to the separation of the southern *P. cinerea* haplogroup (haplogroup B) and later on also to the divergence of the northern *P. cinerea* haplogroup (haplogroup A) and *P. nemaeus*. Subsequently, both populations of *P. cinerea* and *P. nemaeus* came into secondary contact and exchanged again genetic material.

By analyzing sequence data from complete mitochondrial genomes and 12 nuclear loci from all ten odd-nosed monkey species and major haplogroups, the present study provides new and comprehensive insight into the evolutionary and biogeographic history of this enigmatic primate group. Our study supports the hypothesis that odd-nosed monkeys originated on the Asian mainland and migrated into Indochina and Sundaland during the late Miocene. Differentiation into species started in the early Pleistocene, but secondary gene flow among various lineages occurred until the middle Pleistocene. The odd-nosed monkeys as a taxonomic group provide another example that our knowledge of primate diversity is still incomplete (two taxa have been newly described within the last 15 years) and that our understanding of diversification processes is limited. In particular, the impact of secondary gene flow and hybridization seems to be underestimated. The analysis of additional nuclear loci and a population genetic approach including more samples from each taxon will shed further light on the underlying speciation processes in this primate group.

## Materials and Methods

### Ethics statement

Our work was conducted according to relevant German and international guidelines, including countries where we obtained and analyzed samples. Fecal samples from captive animals held at the breeding station of Fanjingshan National Nature Reserve (FNNR), the Kunming Institute of Zoology (KIZ), Beijing Zoo and the Endangered Primate Rescue Center (EPRC), and permissions to use them for our study were provided by Yeqin Yang, director of FNNR, Yaping Zhang, president of KIZ, Jinyuan Zhang, director of Beijing Zoo and Tilo Nadler, director of the EPRC. Fecal samples were collected by institutional staff in a non-invasive way without disturbing, threatening or harming the animals during daily enclosure cleaning. Dried skin material from *R. strykeri* was obtained from a museum specimen described earlier [7] and collection adhered to the legal requirements of Myanmar. Collection of samples adhered to the American Society of Primatologists (ASP) Principles for the Ethical Treatment of Non Human Primates (see www.asp.org/society/resolutions/EthicalTreatmentOfNonHumanPrimates.cfm).

### Sample collection

We obtained fecal samples from captive but wild-born *R. roxellana*, *R. brelichi*, *R. bieti*, *P. cinerea* and *P. nigripes* from the breeding station of FNNR, KIZ and Beijing Zoo, all China, and the EPRC, Vietnam (Table S2). From *R. bieti* and *P. cinerea*, representatives of both major mitochondrial haplogroups (A, B) ([20], [22], [23], Roos unpublished) were sampled. Fresh fecal samples were preserved in 90% ethanol and stored at room temperature before further processing. From *R. strykeri*, we obtained a dried skin fragment from the holotype (AIMZ 15504) housed at the Anthropological Institute and Museum of the University of Zurich (AIMZ), Switzerland [7]. This specimen was collected on March 7, 2010 from two hunters from Pade village (Kachin State, Myanmar; 26.424861°N, 98.312371°E) who had caught the monkey one or two days before [7]. Orthologous sequences of the remaining species (*R. avunculus*, *P. nemaeus*, *N. larvatus*, *S. concolor*) were recently published by our group [14].

### Laboratory methods

DNA from fecal and tissue material was extracted using the QIAamp DNA Stool Mini and DNeasy Blood & Tissue kits from Qiagen following recommendations of the supplier. Complete mitochondrial genomes and nuclear loci were amplified and sequenced using methods outlined in Roos et al. [14]. Due to degradation of DNA extracted from the fecal and dried skin material, mitochondrial genomes were amplified via five overlapping fragments each with a size of ∼5,000 bp. Amplification of nuclear loci was conducted in fragments of less than 1,000 bp [14]. Nuclear markers included five autosomal loci (intron 3 of the serum albumin gene, ALB3; intron 3 of the interstitial retinol-binding protein, IRBP3; intron 1 of the transition protein 2, TNP2; intron 1 of the transthyretin gene, TTR1; intron 11 of the von Willebrand Factor, VWF11), a fragment of the X chromosomal Xq13.3 region and six Y chromosomal loci (intron 5 of the Dead Box gene, DBY5; introns 7 and 11 of the SMC mouse homologue, SMCY7, SMCY11; the SRY gene, SRY; intron 18 of the ubiquitous motif gene, UTY18; last intron of the Zinc finger gene, ZFYLI). Sequences were assembled with GeneiousPro 5.4 [45] and mitochondrial genomes were annotated with the online program DOGMA [46]. Sequences were deposited at GenBank (for accession numbers see Table S2).

### Statistical analysis

For phylogenetic reconstructions, we added further orthologous sequences deposited in GenBank (Table S2). For all loci, the final datasets comprised 24 sequences including 12 odd-nosed monkeys, four other colobines (*Presbytis melalophos*, *Semnopithecus entellus*, *Trachypithecus obscurus*, *Colobus guereza*), five cercopithecines (*Papio hamadryas*, *Theropithecus gelada*, *Macaca sylvanus*, *M. mulatta*, *Chlorocebus aethiops*), and three hominoid species (*Homo sapiens*, *Pan troglodytes*, *Pongo abelii*), which were used as outgroup taxa. All alignments were generated with MAFFT 6 [47] and corrected by eye. In all alignments, poorly aligned positions and indels were removed with Gblocks 0.91b [48] using default settings (Table S1). For the mtDNA, a second alignment (mtDNA2), including only the 12 protein-coding genes on the heavy strand, was generated in Mesquite 2.75 [49].

For phylogenetic analysis, nuclear sequence data were combined, because single loci provided only limited resolution in an earlier study [14] and partition homogeneity tests in PAUP 4.0b10 [50] with 1,000 replications revealed no significant difference in their evolutionary history (see Results). In contrast, mitochondrial and nuclear sequence data were not combined, because partition homogeneity tests suggested that both datasets track different evolutionary histories (P = 0.001, P = 0.002) and because of known incongruent phylogenetic positions of the three langur genera *Semnopithecus*, *Trachypithecus* and *Presbytis* among Asian colobines in mitochondrial and nuclear phylogenies [14].

Phylogenetic trees were constructed with ML and Bayesian algorithms, using the programs GARLI 2.0 [51] and MrBayes 3.1.2 [52], [53]. For all reconstructions, the optimal nucleotide substitution model for each locus was chosen using the Bayesian information criterion (BIC) as implemented in jModeltest 0.1 [54] (Table S1). For phylogenetic analysis, the datasets were whenever appropriate partitioned treating each locus separately and each with its own substitution model. The solely protein-coding alignment of the mitochondrial genome (mtDNA2) was further partitioned into codon positions. In GARLI, only the model specification settings were adjusted, while all other settings were left at their default value. Relative support of internal nodes was assessed by bootstrap analyses with 1,000 replications and ML majority-rule consensus trees were calculated in PAUP. For Bayesian analyses, we used four independent Markov Chain Monte Carlo (MCMC) runs with the default temperature of 0.2. Four repetitions were run for 10 million generations with tree and parameter sampling occurring every 100 generations. Acceptance rates were in the optimal range of 10–70%. The first 25% of samples were discarded as burn-in, leaving 75,001 trees per run. The adequacy of this burn-in and convergence of all parameters was assessed by examining the uncorrected potential scale reduction factor (PSRF) [55] as calculated by MrBayes, which should approach 1 as runs converge and by visual inspection of the trace of the parameters across generations using the software TRACER 1.5 [56]. AWTY [57] was used to check whether posterior clade probabilities were also converging. Posterior probabilities for each split and a phylogram with mean branch lengths were calculated from the posterior density of trees. Alternative phylogenetic relationships among odd-nosed monkey species were tested with the Kishino-Hasegawa test [58] with full optimization and 1,000 bootstrap replications in PAUP.

To estimate divergence ages from the nuclear and mtDNA2 datasets, we applied a Bayesian MCMC method, which employs a relaxed molecular clock approach [59] as implemented in BEAST 1.6.1 [60]. Therefore, we assumed a relaxed lognormal model of lineage variation and a Birth-Death Process prior for branching rates.

As calibrations we used the fossil-based divergence between *Homo* and *Pan*, which has been dated at 6–7 Ma [61]–[63], the separation of *Pongo* from the *Homo*+*Pan* lineage ∼14 Ma [64], the split between *Theropithecus* and *Papio* ∼4 Ma [65], [66], the split between *M. sylvanus* and *M. mulatta* ∼5.5 Ma [67] and the divergence of hominoids and cercopithecoids 24–29 Ma [68], [69]. Instead of hardbounded calibration points, we used the published dates as a normal distribution prior for the respective node. For the *Homo*−*Pan* divergence, this translates into a normal distribution with a mean of 6.5 Ma and a standard deviation (SD) of 0.5 Ma, for the separation of *Pongo* from the *Homo*+*Pan* clade into a mean of 14.0 Ma and a SD of 1.0 Ma, for the *Theropithecus*–*Papio* split into a mean of 4.0 Ma and a SD of 0.5 Ma, for the split between *M. sylvanus* and *M. mulatta* into a mean of 5.5 Ma and a SD of 0.5 Ma, and for the hominoid – cercopithecoid divergence into a mean of 26.5 Ma and a SD of 2.5 Ma.

In BEAST, four replicates were run for 10 million generations with tree and parameter sampling occurring every 100 generations. The adequacy of a 10% burn-in and convergence of all parameters were assessed by visual inspection of the trace of the parameters across generations using TRACER. Subsequently, the sampling distributions were combined (25% burn-in) using the software LogCombiner 1.6.1 and a consensus chronogram with node height distribution was generated and visualized with TreeAnnotator 1.6.1 and FigTree 1.3.1 [70].

## Supporting Information

Table S1 **Locus-specific information including alignment length, number of variable and parsimony-informative sites, and selected substitution models.**
(DOC)

Table S2 **Origin and GeneBank accession numbers of studied species.**
(XLS)
