# Supplementary material for: Evolutionary History of the Odd-Nosed Monkeys and the Phylogenetic Position of the Newly Described Myanmar Snub-Nosed Monkey Rhinopithecus strykeri
Source: PLoS One. 2012 May 16;7(5):e37418. doi: 10.1371/journal.pone.0037418 (PMC3353941; doi:10.1371/journal.pone.0037418)
Supplement: Table S1 — Locus-specific information including alignment length, number of variable and parsimony-informative sites, and selected substitution models. (DOC) [file pone.0037418.s001.doc]

**Table S1.** Locus-specific information including alignment length, number of variable and parsimony-informative sites, and selected substitution models

| **Locus** | **Alignment with / without indels** | **Variable sites / parsimony-informative sites** | **Substitution model (BIC)** |
| --- | --- | --- | --- |
| ALB3 | 1199 / 1149 | 171 / 101 | TPM2uf |
| IRBP3 | 1612 / 1523 | 227 / 124 | HKY + G |
| TNP2 | 899 / 653 | 109 / 64 | TIM3 |
| TTR1 | 906 / 889 | 136 / 85 | TPM3uf |
| vWF11 | 943 / 890 | 157 / 105 | HKY + G |
| **autosomal loci combined** | **5559 / 5104** | **800 / 479** | **-** |
|  |  |  |  |
| **Xq13.3** | **5060 / 4177** | **529 / 355** | **TPM3uf + I** |
|  |  |  |  |
| DBY5 | 1044 / 635 | 138 / 83 | TPM3uf + G |
| SMCY7 | 463 / 404 | 89 / 60 | HKY |
| SMCY11 | 592 / 524 | 103 / 53 | TPM2uf |
| SRY | 786 / 768 | 116 / 71 | K80 |
| UTY18 | 883 / 814 | 135 / 78 | HKY + G |
| ZFYLI | 714 / 676 | 106 / 64 | TPM3uf |
| **Y chromosomal loci combined** | **4482 / 3821** | **687 / 409** | **-** |
|  |  |  |  |
| **nuclear loci combined** | **15101 / 13102** | **2079 / 1243** | **-** |
|  |  |  |  |
| **mitochondrial genome** | **16920 / 15617***  **16920 / 10851**** | **7309 / 5899***  **5535 / 4601**** | **TPM2uf + I + G**  ******* |
|  |  |  |  |
| **all data combined** | **32021 / 28719***  **32021 / 23953**** | **9388 / 7142***  **7614 / 5844**** | **-**  **-** |

* mitochondrial alignment excluding indels and poorly aligned positions (mtDNA1)

** mitochondrial alignment with only the 12 protein-coding genes on the heavy strand (mtDNA2)

*** ND1: HKY+I+G, ND2: TrN+I+G, COI: HKY+I+G, COII: HKY+I+G, ATP8: TrN+I+G, ATP6: TrN+G, COIII: HKY+I+G, ND3: HKY+I+G, ND4L: HKY+I+G, ND4: TrN+I+G, ND5: TrN+I+G, cytb: HKY+I+G
